# Issues on peritoneal metastasis of gastric cancer: an update

**DOI:** 10.1186/s12957-019-1761-y

**Published:** 2019-12-11

**Authors:** Zhen Wang, Jun-qiang Chen, Jin-lu Liu, Lei Tian

**Affiliations:** Department of Gastrointestinal Surgery, The First Affiliated Hospital of Guangxi Medical University, 6 Shuangyong Road, Nanning, 530021 Guangxi Zhuang Autonomous Region China

**Keywords:** Gastric cancer, Peritoneal metastasis, Diagnosis, Intraperitoneal chemotherapy, Conversion therapy

## Abstract

**Background:**

Peritoneal metastasis (PM) is one of the most common forms of metastasis with a very poor prognosis in patients with gastric cancer (GC). The mechanisms, diagnosis, and management of PM remain controversial.

**Main body:**

Stephen Paget’s “seed-and-soil” hypothesis gives us an illustration of the mechanisms of PM. Recently, hematogenous metastasis and exosomes from GC are identified as novel mechanisms for PM. Diagnostic accuracy of conventional imaging modalities for PM is not satisfactory, but texture analysis may be a useful adjunct for the prediction of PM. Biological markers in peritoneal washings are helpful in identifying patients at high risk of PM, but many limitations remain to be overcome. Response of PM from systemic chemotherapy alone is very limited. However, conversion therapy is confirmed to be safe and able to prolong the survival of GC patients with PM. As an important part of conversion therapy, intraperitoneal chemotherapy with taxanes has become an ideal approach with several advantages. Additionally, gastrectomy should be considered in patients who would tolerate surgery if a remarkable response to chemotherapy was observed.

**Conclusion:**

Texture analysis is a reliable adjunct for the prediction of PM, and conversion therapy provides a new choice for GC patients with PM. The underlying mechanisms and new biological markers for GC patients with PM should be the direction of future studies. Furthermore, significant aspects of conversion therapy, such as timing and method of the operation, and the indications remain to be clarified.

## Introduction

Gastric cancer (GC) remains the fourth most common cancer and the second leading cause of cancer-related deaths worldwide, although its incidence and mortality have decreased over the last decades. The mortality rate of GC is highest in East Asia, including China [1]. Generally, most GC patients are diagnosed as advanced stage except countries with national screening programs such as South Korea and Japan, because early-stage GC is commonly asymptomatic [2]. Therefore, the prognosis of patients with GC remains poor, and the 5-year overall survival rate for all the patients diagnosed with GC is only 40–60% in Asia and 24.5% in Europe [3, 4].

Among advanced GC cases, peritoneal implantation is one of the most debilitating and most common forms of metastases. It was reported that peritoneal dissemination rate of GC patients was about 14% at initial examination and the median survival time was 3–6 months [5]. Peritoneal metastasis (PM) of GC was regarded as a terminal disease until the early 1990s because it was considered as unresectable and response from systemic chemotherapy was very limited [6]. However, in the late 1990s, conversion therapy was recommended as a new treatment strategy for PM, with the purpose of en bloc resection of macroscopically detectable lesions by gastrectomy, lymphadenectomy, and peritonectomy, as well as complete elimination of peritoneal micrometastasis by perioperative (including adjuvant and neoadjuvant) chemotherapy (PIC) [6].

In this review, we will discuss the clinical application of conversion therapy for GC with PM, as well as the prediction and diagnosis of PM based on our experience and literatures.

## Mechanisms of peritoneal metastasis

To date, the management of PM of gastric cancer is a challenge for clinicians. Therefore, studying the underlying mechanisms of PM is needed for effective treatment and improving the prognosis of GC patients. Despite Stephen Paget’s 126-year-old “seed-and-soil” hypothesis, insufficient progress has been made towards illuminating the mechanisms governing organ-specific metastasis [7]. This hypothesis compares the optimal microenvironment of the metastatic sites to “soil” and the viable cancer cells to “seeds” [8].

**Fig. 1:**
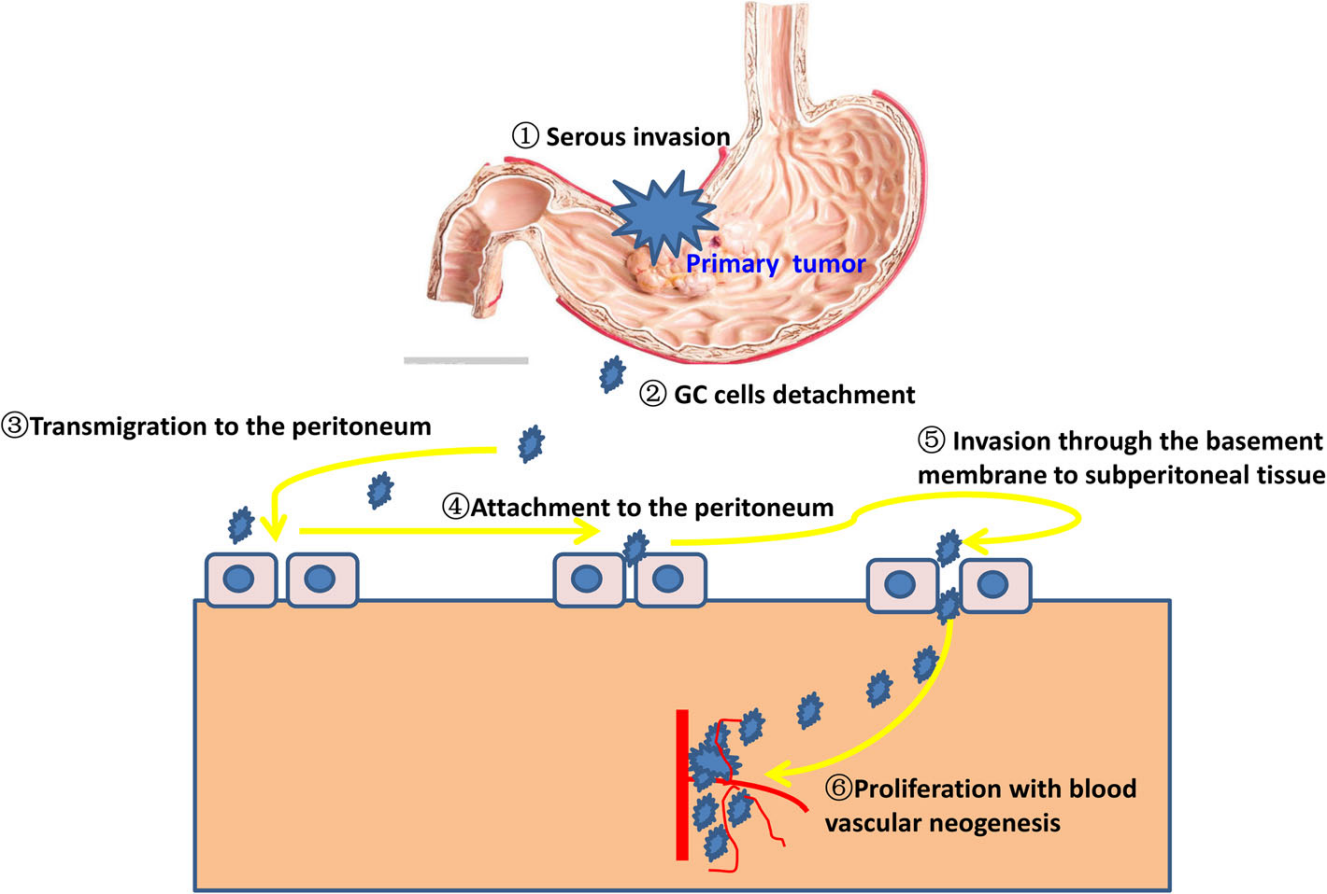
Peritoneal dissemination model of gastric cancer

Based on Paget’s “seed and soil” theory, several consecutive procedures take part in the development of PM of GC, including penetration of tumor tissues through the serous layer, detachment from the primary sites, seeding and survival of the tumor cells within the cavum abdominis, adhesion of tumor cells to the peritoneum, invasion of tumor cells through the basement membrane to subperitoneal tissue, and proliferation with blood vascular neogenesis [8]. In fact, the essence of PM of gastric cancer is the process of cell migration, adhesion, and invasion as well as of epithelial-mesenchymal transition (EMT) and angiogenesis [9]. A number of molecules and signal pathways are associated with this process. Sakakura et al. analyzed a global expression profile (21168 genes) in a cell line that originated from primary GC and other cell lines that originated from metastatic tumor of the cavum abdominis. As a result, they found that 24 genes associated with cell adhesion (such as Integrin alpha3), drug metabolism (such as Aldo-keto reductase family 1), and signal transduction (such as CD9) were upregulated, and 17 genes of immune response (such as CD4) and cell cycle (such as nucleobinding 2) were downregulated [10]. Additionally, studies reported that EMT, which was initiated because of decreased expression of E-cadherin (a membrane glycoprotein), played an important role in PM of gastric cancer [9]. Recently, researchers found that GC-derived exosomes promoted PM by destroying the mesothelial barrier, implicating the crucial role of exosomes in remodeling the premetastatic microenvironment. This identified a novel mechanism for PM of GC [11]. On the contrary, Ge et al. considered that hematogenous metastasis might be the real way of peritoneal implantation by analyzing 5 cases of GC with mesentery dissemination of the small intestine [12]. Therefore, the mechanisms governing the occurrence of PM of GC remain poorly elucidated (Fig. 1).

## Diagnosis and prediction of peritoneal metastasis

Missed preoperative diagnosis of PM is the main reason of surgical failure, poor prognosis, and futile laparotomy in patients with GC. Therefore, accurate preoperative diagnosis of PM, including intraperitoneal free tumor cells and peritoneal dissemination, is especially crucial for prognostic evaluations and alternative therapeutic methods. Imaging examinations such as endoscopic ultrasonography (EUS) and computed tomography (CT) are the common tools for the staging of GC. Nevertheless, we found that EUS is the most sensitive imaging modality with a sensitivity of 0.34 (95% CI, 0.10–0.69) in the diagnosis of PM of GC, which indicated that the accuracy of these imaging modalities was not satisfactory [13].

Texture analysis, which is able to be used in different imaging methods including positron emission tomography, magnetic resonance imaging, CT, and radiography, is a noninvasive imaging modality with quantification of tumoral heterogeneity by assessing spatial variation in gray-level intensities in images [14]. Recently, Kim et al. [15] found that GC patients with peritoneal metastasis showed significantly higher entropy while regarding the texture features. When 7.141 was used as the cut-off value of entropy in the validation study, specificity and sensitivity for the diagnosis of PM were 90% and 80%, respectively. This showed that for GC patients whose PM cannot be found by routine imaging methods such as ultrasound and magnetic resonance imaging, texture analysis might be a useful detection tool.

Intraperitoneal free cancer cells are another type of PM. Many biological markers were studied to improve the diagnostic accuracy of PM. For example, Satoh Y et al. [16] found that evaluation of the expression of MUC2, FABP1, and CK20 by using reverse transcriptase-polymerase chain reaction in peritoneal washings is a useful adjunct in identifying patients at high risk of PM. However, there are still some limitations to overcome before the genetic detection of free tumor cells can be regarded as routine assay. For example, there is no standard method for genetic detection and processing of peritoneal fluid. To make it a reliable detection instrument for free tumor cells, the standard method and process for genetic detection with peritoneal washes should be formulated, and the development of easily available kits and simple diagnostic devices are necessary [17].

Diagnostic laparoscopy is a recommended tool for the staging of GC and offers the indication for a radical surgery. However, existing guidelines vary and make an ambiguity of indication for diagnostic laparoscopy. In order to evaluate the indication and role of diagnostic laparoscopy in the diagnosis of PM, Li et al. [18] conducted a prospective study consisting of 249 cM0 GC patients who underwent diagnostic laparoscopy, and found that tumor-occupied portions (≥ 2 portions) and depth of tumor invasion (≥ 21 mm) of the stomach are predictive factors of PM. Recently, they also registered a new clinical trial to find out the specific indication of diagnostic laparoscopy for Chinese patients [19]. In addition, Song et al. analyzed the clinicopathological data of 163 patients undergoing radical operation for GC and found that the positive rate of the second station of lymph node was an independent risk factor of positive exfoliative cells in peritoneal lavage for GC patients [20]. Moreover, Zhao et al. established an evaluation model consisting of Lauren classification, CA125, CA72-4, and NLR (neutrophil/lymphocyte ratio), and they reported that the evaluation model can effectively predict the risk of PM in GC [21].

These studies provided us useful information to diagnose the PM and to identify the risk factors of PM in GC patients. However, more studies with larger sample size should be helpful in verifying the conclusions.

## Prevention of peritoneal metastasis

Generally speaking, it is accepted that PM is achieved by the implantation of peritoneal free tumor cells. Hence, to eliminate the peritoneal free tumor cells before the actual achievement of PM, taking some effective measures is justifiably considered to be feasible. Based on this view, Masuda et al. investigated the effects of extensive intraoperative peritoneal lavage (EIPL) therapy on advanced GC patients receiving potentially radical gastrectomy. The EIPL procedure was performed by using 1 L physiological saline for 10 times. As a result, they found that EIPL could decrease the peritoneal recurrence of GC [22]. Recently, Kawamura et al. successfully introduced the B4GALNT2 gene (a glycosyltransferase that catalyzes Sd^a^ carbohydrate synthesis) into a human gastric cancer cell line KATO III in vitro by establishing a fiber-modified adenovirus (Ad) vector. And they found that implantation of Ad5/3-B4GALNT2 vectors into the abdominal cavity of mice after inoculation of KATO III cells by surgery could reduce the incidence of PM significantly, which indicated that delivery of a single gene encoding B4GALNT2 modified carbohydrate chains of tumor cells in vivo and reduced cancer metastasis and dissemination. Although the result is based on animal experiment, it provides us a new direction in preventing PM of GC [23].

## Conversion therapy of gastric cancer patients with peritoneal metastasis

Although the improvement of prognosis of GC with PM is achieved by new molecular targeting and chemotherapeutic agents, the therapeutic effect remains unsatisfactory. Multidisciplinary management combining surgery and chemotherapy is believed to become a hopeful therapy because metastatic lesions shrink considerably or disappear apparently after chemotherapy in some cases. Nevertheless, compared with chemotherapy alone, the therapeutic modality with gastrectomy and postoperative chemotherapy failed to offer a survival advantage, which is possibly due to damaged adherence to chemotherapy after surgery [24]. Oppositely, a new multidisciplinary model—conversion therapy, defined as a surgical intervention followed by chemotherapy to cancers that were initially considered as only marginally resectable or unresectable, aiming to achieve an R0 resection [25], is reported to be safe and able to prolong the survival of GC patients with PM [26].

The median overall survival (OS) is only 3–6 months for GC patients with PM without any treatment [27]. Platinum or 5-FU-based regimens have been recommended as the first-line chemotherapy for GC with PM [28]. Nonetheless, the 1-year OS rate is only 16–40.7% and the median OS is as short as 3.1–10.6 months, suggesting the effect of systemic chemotherapy alone is limited [29]. Recently, conversion therapy combining induction chemotherapy and a second surgery seems to give us exciting results. In 2008, Ishigami et al. included 18 stage IV GC patients whose distant metastases were inoperable and treated them with combination chemotherapy of biweekly paclitaxel (PTX) and S-1. Nine of the 18 patients had peritoneal metastasis. After chemotherapy for an average of 6 courses, eight were confirmed as peritoneal dissemination-negative during operation, and the R0 resection rate reached up to 88.9% [30]. Subsequently, Okabe et al. treated 41 GC patients with PM by using chemotherapy with S-1 plus cisplatin [31]. After two cycles of chemotherapy, 19 patients (46%) achieved complete response of peritoneal metastasis, and 22 patients (57.9%) received R0 resection. In the 22 patients who received R0 resection, the 3-year survival rate was 58.4%, with median survival time of 43.2 months, which was significantly longer than for those with noncurative resection (12.6 months) or without surgery (10.3 months) (*P* < 0.0001). After that, many studies evaluated the effect of conversion therapy in GC patients PM, and most of them found that the effectivity of conversion therapy was much better than that of systematic chemotherapy alone [32–41]. However, most of the patients harbored unresectable metastatic lesions including PM, and the survival analysis specifically for GC with PM could not be performed because of confounding data. Therefore, studies focused on GC with PM and subgroup analysis specifically for GC patients with PM is needed in the future (Table 1).

**Table 1:** The clinical application of systematic chemotherapy plus surgery for gastric cancer patients with peritoneal metastasis

Authors reference	Year	Country	Patients	N of PM	Systemic therapy	MST (m)	1-year OS (%)	R0 resection (%)
Ishigami et al. [30]	2008	Japan	Stage IV GC	9	PTX+S1	--	--	88.9
Okabe et al. [31]	2009	Japan	GC patients with PM	41	S-1 plus DDP	43.2	100	57.9%
Einama et al. [32]	2017	Japan	Stage IV GC	3	S1 + DDP/DTX	14.3	100	100
Kanda et al. [33]	2012	USA	GC with PM	19	5-Fu+ oxaliplatin	30.2	94.7	26.3
Han et al. [34]	2013	Korea	M1 GC	8	FOLFOX/DCF	-	-	50
Fukuchi et al. [35]	2018	Japan	Unresectable GC	44	SP et al	-	-	38.6
Satoh et al. [36]	2012	Japan	Stage IV GC	17	S-1 plus DDP	43.5	-	82.4
Sato et al. [37]	2017	Japan	Unresectable metastatic GC	33	DCS	28	97	27.3
Yamaguchi et al. [38]	2018	Japan	Stage IV GC	115	SP et al	-	-	-
Beom et al. [39]	2018	Korea	Stage IV GC	33	Platinum+5-FU	-	-	-
Ramos et al. [40]	2019	Brazil	Unresectable GC	1	XP et al	-	-	-
Kim [41]	2014	Korea	GC patients with PM	43	5-Fu/titanium silicate-1+DDP	37	-	23.3

*DDP*, cisplatin; *DTX*, docetaxel; *HIPEC*, hyperthermic intraperitoneal chemoperfusion; *MMC*, mitomycin C; *PTX*, paclitaxel; *FOLFOX*, 5-FU, oxaliplatin and leucovorin; *DCF*, docetaxel, cisplatin, and 5-FU; *SP*, S-1 plus paclitaxel; *DCS*, docetaxel, cisplatin, and S-1; *XP*, capecitabine and cisplatin

In fact, both the classification of stage IV GC and PM of GC are controversial, which increases the heterogeneity of different studies. As for stage IV GC, Kazuya et al. introduced a new classification system, which is primarily based on the absence (categories 1 and 2) or presence (categories 3 and 4) of macroscopic peritoneal dissemination [38]. As for PM of GC, the TNM classification of The International Union Against Cancer (UICC) is the mainly used classification system, which is consistent with the Japanese classification of gastric carcinoma now [42]. Recently, Fujimura et al. developed a new semi-quantitative scoring system for PM [43]. However, as to which classification system is most helpful in guiding therapy and predicting the prognosis of GC, we have no answer. Therefore, a uniform classification system of PM is urgently needed.

Although results of conversion therapy seem exciting, some researchers still have some doubts to systematic chemotherapy: (1) For GC patients with PM, measurable lesions which are needed in clinical trials to test new anti-cancer agents are very rare, so chemotherapeutic drugs specifically targeting PMs have not been developed (aside from a few exceptions [44]); (2) due to the plasma–peritoneum barrier, which prevents a high concentration of intravenous chemotherapeutic agents from penetrating PM lesions in high concentrations, systemic chemotherapy may not be the best choice for GC patients with PM. Accordingly, another approach, intraperitoneal (IP) chemotherapy, comes to clinicians’ notice.

Recently, researches on the IP administration of chemotherapeutic agents have also demonstrated encouraging progression [45]. IP chemotherapy is an ideal method because of several advantages: (1) Drugs perfused intraperitoneally function immediately on both metastatic lesions on the peritoneal surface and the free tumor cells in the peritoneal cavity; (2) compared with intravenous chemotherapy, IP chemotherapy generates a higher concentration of drug in the abdominal cavity [46]; and (3) some agents are not easily absorbed into the systemic circulation, causing a prolonged half-life in the abdominal cavity and lower systemic toxicity [27]. Intraperitoneal administration of cisplatin (CDDP) or mitomycin C (MMC) has been demonstrated to prolong the survival of GC patients by preventing PM in the adjuvant or neoadjuvant setting [47]. However, some other studies found that IP chemotherapy with MMC or CDDP did not yield apparent therapeutic effects due to brisk drug absorption through the peritoneum [48]. Oppositely, because of their large molecular weight, absorption of taxanes (such as paclitaxel (PTX) and docetaxel (DTX)) is postponed through the lymphatic system after IP administration. Pharmacokinetic researches have also confirmed prolonged retention of DTX and PTX while they are used intraperitoneally [49]. Therefore, intraperitoneal administration of taxanes has been reckoned as a promising method to eliminate PM of GC because of the ability to penetrate directly into PM lesions [45]. To date, many studies were conducted to verify the efficiency of taxanes in the treatment of GC patients with PM [50]. Yamaguchi et al. [51] recruited 35 GC patients with PM and treated them with PTX systematically and PTX plus S-1 intraperitoneally. As a result, in the seven patients with target lesions, the overall response rate was 71% and the 1-year OS rate was 77.1% (95% confidence interval (CI), 60.5–88.1). Malignant ascites decreased or disappeared in 15 of 22 (68%) patients. This showed that intraperitoneal S-1 with PTX was efficient in patients with GC who have macroscopic peritoneal metastasis.

In fact, clinicians also have disputes about the efficiency of IP PTX on the primary lesion. Hence, combination systemic chemotherapy is postulated to enhance the efficiency of IP PTX regionally as well as control the spread of systemic cancer. And studies confirmed that combination modality including systemic chemotherapy and IP chemotherapy was efficient and safe for GC patients with PM [51, 52]. Capecitabine and S-1 are orally available fluoropyrimidine. Both capecitabine and S-1 were found to be well tolerated and equally active in those patients with advanced GC when combining with oxaliplatin. Because S-1 is not widely available globally, the combination of platinum-based chemotherapy and capecitabine is still the most commonly used treatment modality in patients with advanced GC. Chan et al. [27] evaluated the efficacy and feasibility of combining weekly IP PTX with capecitabine and oxaliplatin (XELOX) in the treatment of GC patients with PM. As a result, peritoneal cytology of 11 patients (64.7%) turned negative, the median OS was 18.8 months, and the 1-year survival rate was 72.2%, which showed that IP PTX and XELOX were effective regimens in GC with PM.

Although high-dose intensity therapy to the abdominal cavity may be achieved by IP chemotherapy, deep penetration of drug into the peritoneal surface is limited. It is said that hyperthermic intraperitoneal chemoperfusion (HIPEC) may alter the membrane permeability of cancer cells to increase uptake of anti-cancer agents, and enhance the penetration distance of chemotherapeutic agents by up to 2 mm [53]. Moreover, the combination of chemotherapeutic agents (such as CDDP and mitomycin C) and hyperthermic therapy have shown synergistic cytotoxicity towards tumor cells. Ni et al. [54] tested the effectiveness of a combination of loco-regional chemotherapy (HIPEC) and systemic chemotherapy (intravenous docetaxel) and found that the treatment protocol was useful and feasible, and gained satisfactory clinical outcomes (complete response or partial response in 73.2% of patients). In previous years, studies from Asian countries such as China and Japan supported the usage of HIPEC and cytoreduction for GC patients with PM [55, 56]. Considering differences in genetic risk, tumor biology, epidemiology, treatment, and screening of GC in Western and Asian populations, Badgwell et al. [57] evaluated the effect of HIPEC with cisplatin 200 mg and mitomycin C 30 mg in GC patients with PM. As a result, they found that HIPEC was well tolerated, with short length of hospital stay and a low incidence of complication. Furthermore, they reported a median OS of 30.2 months in patients with GC metastasis limited to abdominal cavity who received multidisciplinary treatment modality including HIPEC. Laparoscopic surgery is very common because of its minimal invasion now. Theoretically, the penetration distance of anticancer agents from the peritoneal surface is significantly shorter in open HIPEC conducted by laparotomy than in laparoscopic HIPEC (LHIPEC) because intraperitoneal pressure is significantly lower in open HIPEC than in closed HIPEC. Therefore, LHIPEC is thought to be much more efficient than open HIPEC. And the result was confirmed by the study of Yonemura et al. [6], who performed LHIPEC in 53 GC patients with PM, and found that LHIPEC is an effective measure of reducing Peritoneal Cancer Index (PCI) before cytoreductive surgery. Nevertheless, Kitayama et al. [58] considered that HIPEC had the risk of peritoneal adhesion, which may seriously impair the effectiveness of the continued IP chemotherapy, and thus they did not recommend the usage of HIPEC (Table 2).

**Table 2:** The clinical application of combination of IP chemotherapy and systematic chemotherapy for gastric cancer patients with peritoneal metastasis

Authors reference	Year	Country	Patients	N	IP therapy	Systemic therapy	MST (m)	1-year OS (%)	R0 resection (%)
Yamaguchi [51]	2013	Japan	GC patients with PM	35	PTX	PTX+S1	17.6	77.1	60.0
Kitayama [52]	2014	Japan	GC patients with PM	64	PTX	PTX+S1	26.4	82.0	53.1
Badgwell [57]	2017	USA	GC patients with PM	19	HIPEC MMC and CDDP	5-Fu+ oxaliplatin	30.2	94.7	26.3
Yonemura [6]	2017	Japan	GC patients with PM	52	HIPEC DTX and CDDP	DTX, CDDP and S1	19.2	-	57.6

*CDDP*, cisplatin; *DTX*, docetaxel; *HIPEC*, hyperthermic intraperitoneal chemoperfusion; *MMC*, mitomycin C; *PTX*, paclitaxel

Generally speaking, the effect and safety of chemotherapeutic agents are closely associated with the dose. Therefore, the optimal dose should be tested for agents used in IP chemotherapy. In determining the recommended dose (RD) of IP docetaxel, Cho et al. [59] tried IP docetaxel at 3 different dose levels (100, 80, or 60 mg/m^2^). As a result, they found that the RD of intraperitoneal docetaxel (100 mg/m^2^) was effective with manageable toxicities in the treatment of GC patients with PM. With a median follow-up duration of 20.8 months in the surviving patients, the progression-free survival (PFS) rate at 6 months was up to 69.0% (95% CI 53.7–84.3), which exceeded the pre-designed percentage needed to meet the primary outcome of the study. Abdominal pain was the most frequent grade 3/4 non-hematological toxicities of IP chemotherapy. And researchers considered that the frequent grade 3/4 abdominal pain was due to bowel irritation caused by IP chemotherapy [60]. However, it was treatable by dose reduction and the therapy of analgesics in majority of the patients, and none of them discontinued treatment due to bellyache [59].

Gastrectomy was an important part of conversion therapy. However, significant aspects, such as timing and method of the operation, and the indications, remain to be clarified [61]. Generally speaking, gastrectomy was suggested for patients who would tolerate surgery, if an obvious effect of combination chemotherapy was demonstrated. The indications for gastrectomy were no unresectable metastasis observed after imaging examination, the obvious shrinkage or disappearance of peritoneal metastasis, and negative peritoneal cytology findings. Commonly, the response of PM was checked by second-look laparoscopy, the timing of which was determined according to the effect of chemotherapy and the degree of PM before chemotherapy [26].

## Conclusion

Peritoneal implantation is one of the most debilitating and most common forms of metastases of gastric cancer. Besides commonly used tools, such as EUS and CT, genetic testing might be an alternative choice for the diagnosis of PM. However, diagnostic laparoscopy remains the first choice to confirm PM until now. Conversion therapy, which combines surgical intervention with chemotherapy, is confirmed to be efficient for GC patients with PM. With the advantage of large molecular weight, taxane-based IP chemotherapy is postponed through the lymphatic system, which can function on both metastatic lesions on the peritoneal surface and the free tumor cells in the peritoneal cavity. Therefore, conversion therapy combining systematic chemotherapy and IP chemotherapy, followed by surgical intervention, should be the first choice of GC patients with PM. Although great progress has made in terms of classification, diagnosis, and treatment, the management of PM of GC faces a number of problems: (1) Many limitations should be overcome before genetic diagnosis for free tumor cells can be used as routine method; (2) more clinical studies with larger sample size focused on the conversion therapy of GC with PM are needed in future. (3) Significant aspects of conversion therapy, such as timing and method of the operation, and the indications, remain to be clarified.

## Data Availability

Data sharing is not applicable in this article as no datasets were generated or analyzed during the current study.
